# Cincho-Quinine

**Published:** 1875-04

**Authors:** 


Cincho Quinine.—Elsewhere we present this month the qualitative analysis of this educt from Peruvian bark, over the signatures of analytical chemists of undoubted authority. It is satisfactory to know that a remedy which we have for so long time kept prominently before our readers in our advertising columns is not the gross fraud which has been charged.

